# Low back pain in 17 year olds has substantial impact and represents an important public health disorder: a cross-sectional study

**DOI:** 10.1186/1471-2458-12-100

**Published:** 2012-02-05

**Authors:** Peter B O'Sullivan, Darren J Beales, Anne J Smith, Leon M Straker

**Affiliations:** 1 School of Physiotherapy and Curtin Health Innovation Research Institute, Curtin University, GPO Box U1987, Perth, WA 6845, Australia; 2 Telethon Institute for Child Health Research, Perth, Australia

## Abstract

**Background:**

Prevalence of low back pain (LBP) rises rapidly during adolescence, reaching adult levels by the age of 18. It has been suggested that adolescent LBP is benign with minimal impact, despite limited evidence.

**Methods:**

The aim of this study was to investigate the impact of LBP and the influence of chronicity, gender and presence of other spinal pain comorbidities at age 17. Subjects (n = 1283) were categorised according to experiencing current and chronic LBP, gender and presence of other areas of spinal pain. LBP impact was ascertained via questions regarding seeking professional assistance, using medication, missing school/work, limited normal or recreational physical activity and health related quality of life (HRQOL).

**Results:**

12.3% of participants reported current but not chronic LBP, while 19.9% reported current chronic LBP. LBP was more commonly reported by females than males. Other spinal pain comorbidities were common in the LBP groups. Impact was greater in subjects with chronic LBP, in females and in those with other spinal pain comorbidities.

**Conclusion:**

LBP, and particularly chronic LBP, has a significant negative impact at 17 years. It is commonly associated with care seeking, medication use, school absenteeism, and reduced HRQOL. These findings support that adolescent LBP is an important public health issue that requires attention.

## Background

Disabling chronic low back pain (LBP) is one of the greatest adult public health disorders in the industrialised world [1]. For the majority with LBP disorders there is no diagnosis, leaving ~90% classified as non-specific LBP [2,3].

There is growing evidence that LBP commonly develops in adolescence with prevalence rates reaching towards adult rates by late adolescence [4,5]. Gender differences in LBP prevalence, observed in adults, also emerge during adolescence [6,7]. Furthermore adolescent LBP is a strong predictor of adult LBP [8,9] suggesting adolescent LBP may set a course for later life.

It is not clear whether the burden commonly associated with adult LBP is present during adolescence as the specific impact of adolescent LBP has not been well investigated. Some studies have reported that adolescent LBP is associated with seeking professional help [10,11], using medications [10], school absenteeism [12], reduced activity levels [11-13] and reduced health related quality of life (HRQOL) [10]. However these studies have had limitations. For example, although Harreby et al. (1999) reported that 15.5% of LBP subjects had consulted a physician for their problem no measure of disability or impact was gained. Disability was assessed by Watson et al. (2002), with 24% of LBP subjects seeking professional help and 94% reporting a degree of impairment. However this study was criticised for overstating the problem because of poor levels of child-parent agreement [14].

Recently 39.8% of a 15 year old cohort reported LBP lasting at least one day in the last month, and this was associated with only low levels of disability and no difference in HRQOL from those without pain, except when it was associated with whole body pain [15]. These authors concluded that localised LBP is a normal life experience during adolescence with negligible impact, and should not be given significant attention as a health disorder [15]. It has been similarly proposed by others that medicalisation of this disorder in adolescence may be detrimental, leading to pain behaviours and poor coping strategies that result in increased burden [14]. However these conclusions are based on a study of 15 year olds that did not consider chronicity of LBP, nor assess impact across a broad range of variables.

The aim of the present study was to characterise the burden of LBP in 17 year olds in terms of specific LBP related impact (regarding health care utilisation, work and/or study absenteeism and activity avoidance) and general HRQOL. This addresses some of the limitations in the current literature regarding the impact of LBP in adolescents. It was hypothesised that LBP in 17 year olds would be associated with significant impact and reduction in HRQOL, and that this would be greater in LBP that was chronic, in females and when associated with other areas of spinal pain.

## Methods

### Participants

Cross-sectional data were obtained from adolescents aged (mean (standard deviation)) 17.0 (0.3) years, 52.8% who were female, participating in the West Australian Pregnancy Cohort "Raine" Study (http://www.rainestudy.org.au). This long-term project began as a pregnancy cohort in which 2,900 women attending antenatal clinics at a tertiary level obstetric hospital in Perth, Western Australia were enrolled between 1989 and 1991. 2,868 children born to 2,804 mothers remained with the study to form the Western Australian Pregnancy Cohort (Raine) Study. 1475 of the original participants completed some aspect of the 17 year follow-up (three questionnaires and a physical examination), and 1307 (88.6%) completed the paper questionnaire covering LBP prevalence, LBP impacts, and the SF-36 Version 1 Health Survey. A comparison of the Raine cohort at birth compared to the general Western Australian population using the Western Australian Maternal and Child Health Research Database found the Raine cohort to be at higher risk in terms of prenatal and perinatal characteristics and to be overly representative of socially disadvantaged families [16], whilst a comparison of the characteristics of participants versus non-participants 14 years after cohort inception found that socially disadvantaged families were less likely to remain in the cohort to age 14 years [17]. However, when participating families in the 17 year follow-up were compared to the Western Australian population of families with 15 to 17 year old children it was found that the Raine sample had a lower proportion of rural dwelling families (18.4% versus 33.9%, *p *< 0.001), a slightly higher proportion of urban dwelling families in high socioeconomic status neighbourhoods (23.6% versus 20.6%), and a slightly lower proportion of families with a combined family income of less than AUS$25,000 (7.9% versus 10.8%). Ethnicity in the cohort is predominantly Caucasian (93%). Ethical approval was granted from Curtin University Human Research Ethics Committee and the West Australian Department of Health Ethics Committee. Consent was obtained from the adolescents' guardians.

### Measures

The prevalence of LBP and its impact, and the presence of mid-back pain (MBP) and neck and shoulder pain (NSP), were ascertained from the questions in Table 1, which along with the SF-36 Version 1 [18] were administered as part of a larger questionnaire examining multiple lifestyle and medical dimensions. 1246 subjects provided valid SF-36 and pain data, while 1283 subjects provided valid impact and pain data. MBP and NSP were included in the catagorisation of pain given the potential significant impact that multiple bodily pain areas may have on the interpretation of LBP related impact [15,19].

**Table 1:** Questions related to the prevalence and impact of low back pain

Prevalence Questions:	
P1	Has your low back been painful at any time in the last month?

P2	Has your low back pain ever lasted for more than 3 months off and on (it hurt at least once a week but not every day)?

P3	Has your low back pain ever lasted for more than 3 months continuously (it hurt more or less every day)?

P4	Has your midback been painful at any time in the last month?

P5	Has your neck/shoulder been painful at any time in the last month?

**Impact Questions:**	

I1	Have you ever sought health professional advice or treatment for low back pain?

I2	Have you ever taken medication to relieve the low back pain?

I3	Have you ever missed school or work due to the low back pain?

I4	Has the low back pain ever interfered with your normal activities?

I5	Has the low back pain ever interfered with recreational physical activities (eg sport, walking, cycling etc.)?

### Pain

The experience of LBP was ascertained via the Nordic LBP questionnaire [20], with modifications based on subsequent research[21-23]. LBP in the sample was characterised by the formation of three mutually exclusive LBP prevalence groups according to answers to questions P1 to 3 in Table 1; i) no current LBP (NO_LBP; 'No' to P1), ii) current non-chronic LBP (CNC_LBP: 'Yes'to P1, and 'No' to P2 and P3) iii) current chronic LBP (CC_LBP: 'Yes'to P1, and 'Yes' to P2 or P3). Pain comorbidities in other spinal regions, regardless of LBP status, was characterised by formation of three mutually exclusive groups according to answers to P4 and P5 in Table 1; i) No MBP or NSP ('No' to P4 and P5) ii) MBP or NSP ('Yes to one only of P4 and P5) and iii) MBP and NSP ('Yes to both P4 and P5).

### Specific impacts of low back pain

LBP impacts were ascertained from questions I1 to I5 in Table 1. These represent previously validated questions that capture different aspects of impact associated with LBP [20,24,25]. In those subjects with current LBP, a count variable indicating number of LBP impacts experienced was also derived.

### Health-related quality of life

The SF-36 is a widely used HRQOL questionnaire [18,26]. This study used the SF-36 Version 1 which has been used in 130 Australian studies and validated in several [27]. The SF-36 is a generic instrument for assessment of HRQOL consisting of 36 items measuring: physical functioning, role physical, bodily pain, general health, vitality, social functioning, role emotional, and mental health. Factor analytic studies in a number of populations have confirmed the existence of two higher order factors representing physical and mental health factors, the Physical Component Summary (PCS) and Mental Component Summary (MCS) measures [28]. PCS and MCS measures were calculated using Australian factor weightings, based on the Australian National Bureau of Statistics 1995 Australian National Health Survey dataset [29] and scored on a 0-100 scale and normalised to have a mean of 50 and a standard deviation of 10, based the 1995 Australian National Health Survey data.

### Data analysis

Chi-squared tests were performed to test associations between LBP group and gender, and LBP group and other spinal pain areas. In those subjects with current LBP only, chi-squared tests were performed to test associations between LBP group and specific LBP impacts, and chi-squared test for linear trend of association between LBP group and count of LBP impact.

In all subjects, differences in LBP groupings between SF-36 health status scores were estimated using linear regression models with SF36 scores as the dependent variable and the three category variable 'LBP group' parameterised by two dummy predictor variables, one representing CC-LBP and one representing CNC-LBP. Final estimates of LBP group differences were adjusted for gender and presence of other spinal pain areas. Statistical significance was adjusted to α = 0.008 to account for multiple tests of 2 outcomes and 3 pain groups. Interactions between gender and LBP group, and between other spinal regions (MBP, NSP) and LBP group were tested with statistical significance at α = 0.05. Model diagnostics confirmed homogeneity of residual variance and absence of influential cases. Statistical analysis was performed with Stata/IC 10.1 for Windows (Statacorp LP, College Station TX).

## Results

Table 2 reports the prevalence of CNC_ LBP and CC_LBP. Females reported CNC_LBP and CC_LBP more than males (Table 2). Proportions of participants with other spinal pain areas were also significantly different between LBP groups (Table 2), with almost half of those participants reporting MBP and NSP also reported CC_LBP.

**Table 2:** Differences in LBP group proportions (n(%)) overall and by gender, presence of other spinal pain areas and specific LBP impacts

	No LBP	Current Non-chronic LBP	Current chronic LBP	
**Overall (n = 1288)**	873 (67.8)	158 (12.3)	257 (19.9)	

**Gender**				

Males (n = 610)	474 (77.7)	54 (8.9)	82 (13.4)	< 0.001

Females (n = 678)	399 (58.9)	104 (15.3)	175 (25.8)	

**Other Spinal Pain areas**				

No MBP or NSP (n = 736)	604 (82.1)	70 (9.5)	62 (8.4)	< 0.001

MBP or NSP (n = 368)	198 (53.8)	63 (17.1)	107 (29.1)	

MBP and NSP (n = 178)	67 (37.6)	25 (14.0)	86 (48.3)	

**Specific LBP Impacts (n ~ 415)**				

Sought health professional care (n = 156 of 415, 37.6%)	-	30 (19.2)	126 (80.7)	0.007

Took medication (n = 144 of 414, 34.8%)	-	42 (29.2)	102 (70.8)	< 0.001

Missed school/work (n = 88 of 414, 21.3%)	-	14 (15.9)	74 (84.1)	< 0.001

Interfered with normal activities (n = 160 of 412, 38.8%)	-	30 (18.8)	130 (81.2)	< 0.001

Interfered with physical activities (n = 179 of 411, 43.6%)	-	43 (24.0)	136 (76.0)	< 0.001

**Count of specific LBP impacts**				

0 (n = 139 of 412, 33.7%))	-	82 (59.0)	57 (41.0)	< 0.001

1 (n = 78, 18.9%)	-	32 (41.0)	46 (59.0)	

2 (n = 58, 14.1%)	-	18 (31.0)	40 (69.0)	

3 (n = 55, 13.4%)	-	10 (18.2)	45 (81.8)	

4 (n = 42, 10.2%)	-	9 (21.4)	33 (78.6)	

5 (n = 40, 9.7%)	-	5 (12.5)	35 (87.5)	

Frequency of LBP related impacts for participants reporting current LBP are shown in Table 2). In those participants reporting current LBP, there were significantly greater proportions reporting each of the LBP specific impacts in the CC_LBP group (*p *< 0.001 to *p *= 0.007, Table 2). There was also a linear association between the number of reported impacts and membership of the CC-LBP group (*p *< 0.001, Table 2).

Table 3 presents the means and standard deviation for SF-36 PCS and MCS scores separately by LBP group, gender, and other areas of spinal pain. Those subjects with CC_LBP had significantly lower PCS and MCS scores than those subjects with CNC-LBP (*p *< 0.001 and *p *= 0.003 respectively) and those subjects with No LBP (*p *< 0.001 for both). Those subjects with CNC_LBP had significantly lower PCS and MCS scores than those subjects with No LBP (*p *< 0.001 for both). This association between poorer PCS and MCS scores was also apparent by examining the proportion of participants with scores less than 40, i.e. more than one standard deviation below the Australian population mean. For PCS scores, these proportions were 40 of 845 (4.7%) in No LBP group, 14 of 156 (9.0%) in CNC_LBP group and 50 of 246 (20.3%) in CC_LBP, which was a statistically significant difference (*p *< 0.001). For MCS scores, these proportions were 84 of 845 (9.9%) in No LBP group, 30 of 156 (19.2%) in CNC_LBP group and 68 of 246 (27.4%) in CC_LBP, which was also a statistically significant difference (*p *< 0.001).

**Table 3:** SF36 Physical and Mental Component Summary Scores (Mean(SD)) by LBP group, gender and other spinal pain areas

	PCS	*p*-value	MCS	*p*-value
**LBP group**				

No LBP	53.2 (6.4)	< 0.001	51.8 (7.9)	< 0.001

Current Non-chronic LBP	50.3 (6.9)^a^		48.5 (9.2) ^a^	

Current chronic LBP	47.4 (9.0)^a, b^		45.8 (10.1) ^a, b^	

**Gender**				

Male	53.6 (6.0)	< 0.001	52.7 (7.4)	< 0.001

Female	50.0 (8.1)		48.0 (9.5)	

**Other Spinal Pain areas**				

No MBP or NSP	53.8 (6.0)	< 0.001	52.3 (7.8)	< 0.001

MBP or NSP	49.7 (8.1)^c^		48.2 (9.6) ^c^	

MBP and NSP	47.1 (8.1)^c, d^		45.6 (9.0) ^c, d^	

^a ^statistically significant contrast to No LBP

^b ^statistically significant contrast to Current Non-chronic LBP

^c ^statistically significant contrast to No Mid Back Pain or Neck/Shoulder Pain

^d ^statistically significant contrast to MBP or NSP

Females displayed significantly lower PCS and MCS scores than males (*p *< 0.001 for both, Table 3). Those subjects with MBP and NSP had significantly lower PCS and MCS scores than those subjects with MBP or NSP (*p *< 0.001 for both) and those subjects with No MBP or NSP (*p *< 0.001 for both, Table 3). Those subjects with MBP or NSP had significantly lower PCS and MCS scores than those subjects with No MBP or NSP (*p *< 0.001 for both, Table 3).

Table 4 presents the results of the two multivariable linear regression analyses for SF-36 PCS and MCS scores, using LBP group, gender and presence of other spinal pain areas as the independent variables, in the 1242 participants with a full and valid set of data for multivariable analysis. The β coefficients represent the estimated mean difference between the reference group and the contrast group, adjusted for the other variables in the model. There was a significant interaction effect between gender and LBP-group for the PCS scores (*p *= 0.003), with females displaying larger and statistically significant differences between LBP groups. In females, a statistically significant difference in scores was estimated between CC_LBP and No LBP (*p *< 0.001, Table 4), and between CC_LBP and CNC_LBP (*p *< 0.001, Table 4). Similarly, a larger gender difference was estimated in CC_LBP than in No LBP or CNC_LBP (Table 4). No interaction between LBP group and other areas of spinal pain was detected, meaning that the pattern of difference in LBP groups was similar irrespective of the presence of pain in other spinal pain areas. However, the presence of pain in other spinal areas was also significantly associated with lower PCS and MCS scores, independently of gender and LBP group (Table 4).

**Table 4:** Regression Coefficients^1 ^for General Linear Model for SF36 Physical and Mental Component Summary Scores (n = 1242)

Physical Component Summary Scores		
	**β (95% CI)^1^**	***p*-value**

**LBP group^2^**		

No LBP	REF	

Current Non-chronic LBP		

Males	-2.16 (-4.09,-0.23)	0.028

Females	-1.39 (-2.85, 0.07)	0.062

Current chronic LBP		

Males	-1.28 (-2.93,0.36)	0.127

Females	-4.60 (-5.88,-3.32)	< 0.001

**Gender^3^**		

No LBP		

Males	REF	

Females	-1.90 (-2.81,-0.99)	< 0.001

CNC LBP		

Males	REF	

Females	-1.13 (-3.36, 1.10)	0.320

CC LBP		

Males	REF	

Females	-5.21(-7.02,-3.40)	< 0.001

**Other SP areas**		

No MBP or NSP	REF	

MBP or NSP	-3.04 (-3.93,-2.16)	< 0.001

MBP and NSP	-4.56 (-5.76,-3.37)	< 0.001

**Mental Component Summary Scores**		

	β (95% CI)	*p*-value

**LBP group**		

No LBP	REF	

Current Non-chronic LBP	-1.75 (-3.19,-0.31)	0.017

Current chronic LBP	-3.45 (-4.73,-2.17)	< 0.001

**Gender**		

Males	REF	

Females	-3.53 (-4.47,-2.58)	< 0.001

**Other SP areas**		

No MBP or NSP	REF	

MBP or NSP	-2.83 (-3.93,-1.74)	< 0.001

MBP and NSP	-4.40 (-5.87,-2.93)	< 0.001

^1^β coefficients represent the estimated mean difference between the reference group and the contrast group, adjusted for the other variables in the model

^2 ^Significant gender × LBP group interaction (*p *= 0.003), therefore effects for LBP group reported separately by each gender, and

^3 ^Significant gender × LBP group interaction (*p *= 0.003), therefore effects for female gender reported separately by each category of LBP group.

Statistically significant differences in MCS scores were observed between LBP groups that were independent of gender and presence of pain in other spinal areas (Table 4). Statistically significant differences in MCS scores were also observed between males and females that were independent of LBP group and presence of pain in other spinal areas (Table 4). Lastly, statistically significant differences in MCS scores were also observed between groups defined by report of pain in other spinal areas that were independent of LBP group and gender (Table 4).

## Discussion

The results of this study suggest that there is substantial burden related to adolescent LBP. This is demonstrated by the findings that in 17 year olds LBP was associated with poorer mental and physical HRQOL. The difference in these aspects of HRQOL of 3 or more points (Table 3) can be considered not just statistically significant but also clinically meaningful [30]. Additionally LBP was associated with specific negative impact across a number of different domains, namely 'care seeking behaviours' (seeking professional care, medication use for symptom control) and 'activity modification behaviours' (school/work absenteeism as well as reduced normal and recreational activities). These are all factors considered important in assessing the impact of pain in adolescents [31]. Furthermore, the impact was more apparent in subjects with chronic LBP than those with non-chronic LBP, in females and in those with other spinal pain comorbidities. Although previous studies have documented adolescent LBP impact across similar domains (seeking professional help [10,11], using medications [10], school absenteeism [12], reduced activity levels [11-13] and reduced HRQOL [10]) the strength of our study is that it investigated impact across these multiple domains simultaneously. The use of multiple LBP impacts allows for counts of number of impacts in individuals (Table 2). From this we ascertained that subjects with CC_LBP more frequently report multiple impacts compared to CNC_LBP, an occurrence that does not appear to have been previously reported in the literature.

The results of our study contrast with those of Pellise et al. (2009) who concluded that adolescent LBP has no significant impact. This was based upon their finding in 15 year olds that HRQOL (assessed with the KIDSCREEN-52) and LBP specific functional scales (Roland-Morris Disability Questionnaire, Hanover Functional Ability Questionnaire) did not differ between subjects with no LBP, LBP, other pain and LBP plus other pain (but not whole body pain). Only when LBP presented as part of a whole body pain were decreased HRQOL and LBP related function reduced [15]. The difference between our results and those of Pellise et al. (2009) could be related to a number of factors. Firstly our study looked at current LBP in the context of chronicity. However 17 year olds in our CNC_LBP group still reported impact from their LBP, although not as marked as in the CC_LBP group. Secondly, our subjects were older (average age of 17, versus 15 in the Pellise et al. (2009) study) suggesting the possibility that impact of adolescent LBP may not become apparent until later in adolescence. Measures of impact and HRQOL in our study may be more sensitive to detect differences in adolescent LBP populations than those investigated by Pellise (2009). Alternately population differences could underlie the differences.

The gender differences observed in the current study highlight the greater prevalence of CNC_LBP, CC_LBP and spinal pain comorbidities in females when compared to males. This is consistent with previous studies of children and adolescents [6,7], young adults [19] and adults [32]. Furthermore females reported poorer physical and mental HRQOL measures than males which has also been found in 19 year olds [19]. Gender differences in pain and its impact are likely to be a complex interaction of genetic, neurophysiological, hormonal and psychosocial factors [33].

Previous research has reported that widespread chronic pain [24,34] and generalised spinal pain (as opposed to localised LBP) [23,35] in adolescents has a negative impact on wellbeing. For example schoolchildren and adolescents with numerous variations of chronic body pain have been reported to have increased school absenteeism, medication use and medical care, restrictions of daily activities and hobbies, and decreased HRQOL [24,34]. Additional impacts in these subjects include sleep disturbance, eating problems and reduced social interaction [34]. Additionally, a recent report shows reduced HRQOL in 19 year olds related to multiple pain regions [19]. Our findings of greater impact in adolescents with comorbid NSP and MBP are consistent with these reports.

The presence of other areas of pain in the current study displayed an additive effect in regression models for PCS and MCS. Therefore, both LBP and other spinal pain areas contribute separately to reduced HRQOL. For example, for females the combination of CC_LBP with the presence of MBP and NSP was estimated to be associated with a mean reduction in PCS of -4.6 plus -4.6, or -9.2 points, compared with no spinal pain in any area, and for males this estimate was -1.3 plus -4.4, or -5.7 points. For MCS scores, the combination of CC_LBP with the presence of MBP and NSP was estimated to be associated with a mean reduction in PCS of -3.4 plus -4.4, or -7.8 points. The absence of an interaction between the two variables means that the estimated decrease in PCS and MCS scores with the presence of other spinal pain areas was of similar magnitude regardless of the presence of CNC_LBP or CC_LBP, and that the estimated decrease in scores with the CNC-LBP or CC_LBP was of similar magnitude regardless of the presence of other spinal pain areas. These findings demonstrate a similar pattern to those reports of greater levels of disability and poorer HRQOL outcomes in adults with increased pain areas [19,36,37].

This study had a limitation in that results may have been influenced by selective attrition in that more socially disadvantaged participants were less likely to remain in the cohort. However, the cohort 17 years after inception appears to be slightly more socially advantaged than the Western Australian population of families with 15-17 year old children. The effect of this bias is likely to be either minimal [38,39], or such that associations detected between pain groups, pain area and gender and impact of pain may be attenuated, given that studies have shown greater impact of pain in adults may be associated with social disadvantaged population [40-42].

## Conclusion

This study supports that LBP is associated with substantial impact in 17 year olds across a number of important domains (LBP related care seeking behaviours, negative activity modification behaviours and poorer physical and mental HRQOL). These impacts may be related to long-term negative consequences. As an example, school absenteeism has been associated with reduced academic achievement, poor social interaction in adolescence and adulthood and reduced participation in the workforce [43,44]. This supports the view that adolescent LBP at 17 years is a significant public health issue that requires attention.

Furthermore these findings in 17 year old adolescents closely reflect the prevalence rates, gender differences, spinal pain comorbidities, impact profiles related to both physical factors and mental health commonly reported in adult LBP populations [3,32] suggesting adolescence may be an important transitional period towards the development of adulthood LBP and associated burden. While the presence of LBP in adolescence is a strong predictor of adult LBP [8,9], future longitudinal research is needed to determine whether the impacts and behaviours of LBP observed during adolescence are predictors for the same in adulthood. The Raine cohort will be tracked into adulthood to assess this premise. Also early life longitudinal studies are need to determine whether the poorer mental and physical HRQOL findings proceed or are a consequence of adolescent LBP.

Further studies are also required to investigate the underlying mechanisms associated with the development of disabling LBP across adolescence [2,3]. Such studies need to recognise the multifactorial biopsychosocial nature of these disorders [3,45,46]. Information from future biopsychosocial mechanistic studies may then be utilised to inform trials of targeted prevention and management interventions at the point of development of these disorders and their associated impact. Developing a greater understanding of adolescent LBP and addressing it could be an effective strategy to reducing the overall burden caused by LBP in society. Targeting adult LBP behaviours in adolescents, before they become entrenched, may prove to be a more successful intervention strategy than those targeting behaviours that are potentially more entrenched by adulthood [47]. Therefore adolescent LBP at 17 years of age should be recognised as an important health issue.

## Competing interests

The authors declare that they have no competing interests.

## Authors' contributions

All authors' contributed equally to this study.

## Authors' information

The authors' represent a group of physiotherapy academics/clinicians dedicated to investigating the development, underlying mechanisms and management of musculoskeletal disorders from a biopsychosocial framework.

## Pre-publication history

The pre-publication history for this paper can be accessed here:

http://www.biomedcentral.com/1471-2458/12/100/prepub
